# A 7-year review of clinical characteristics, predisposing factors and outcomes of post-keratoplasty infectious keratitis: the Nottingham infectious keratitis study

**DOI:** 10.3389/fcimb.2023.1250599

**Published:** 2023-08-30

**Authors:** Zun Zheng Ong, Thai Ling Wong, Lakshmi Suresh, Yasmeen Hammoudeh, Michelle Lister, Dalia G. Said, Harminder S. Dua, Darren S. J. Ting

**Affiliations:** ^1^ Department of Ophthalmology, Queen’s Medical Centre, Nottingham, United Kingdom; ^2^ Department of Microbiology, Queen’s Medical Centre, Nottingham, United Kingdom; ^3^ Academic Ophthalmology, School of Medicine, University of Nottingham, Nottingham, United Kingdom; ^4^ Birmingham and Midland Eye Centre, Birmingham, United Kingdom; ^5^ Academic Unit of Ophthalmology, Institute of Inflammation and Ageing, University of Birmingham, Birmingham, United Kingdom

**Keywords:** keratitis, infectious keratitis, corneal ulcer, corneal infection, microbial keratitis, keratoplasty, corneal transplant, corneal transplantation

## Abstract

**Background/objectives:**

Post-keratoplasty infectious keratitis (PKIK) is a unique sight-threatening clinical entity which often poses significant therapeutic challenges. This study aimed to examine the clinical presentation, risk factors, management, and clinical outcomes of PKIK.

**Methods:**

This was a retrospective study of all patients who presented to the Queen’s Medical Centre, Nottingham, with PKIK between September 2015 and August 2022 (a 7-year period). Relevant data on types of keratoplasty, clinical presentations, causative microorganisms, management, and outcome were analyzed.

**Results:**

Forty-nine PKIK cases, including four cases of interface infectious keratitis, were identified during the study period. The most common graft indications for PKP, DALK and EK were failed grafts (9, 37.5%), keratoconus (6, 54.5%) and Fuchs endothelial corneal dystrophy (FECD; 8, 57.1%), respectively. *Staphylococcus* spp. were the most commonly identified organisms (15, 50.0%). Bullous keratopathy (18, 36.7%), ocular surface disease (18, 36.7%), and broken/loose sutures (15, 30.6%) were the most common risk factors. Concurrent use of topical steroids was identified in 25 (51.0%) cases. Of 31 functioning grafts at presentation, 12 (38.7%) grafts failed at final follow-up with 15 (48.4%) patients retaining a CDVA of ≥1.0 logMAR. The overall estimated 5-year survival rate post-PKIK was 55.9% (95% CI, 35.9%-75.9%), with DALK having the highest survival rate [63.6% (95% CI, 28.9%-98.3%)], followed by EK [57.1% (95% CI, 20.4%-93.8%)] and PKP [52.7% (95% CI, 25.1%-80.3%)], though no statistical difference was observed (p=0.48).

**Conclusions:**

PKIK represents an important cause of IK and graft failure. Bullous keratopathy, OSD and suture-related complications are the commonest risk factors, highlighting the potential benefit of prophylactic topical antibiotics (for unhealthy ocular surface) and early suture removal (where possible) in reducing the risk of PKIK. Graft survival may be higher in lamellar keratoplasty following PKIK but larger studies are required to elucidate this observation.

## Introduction

Approximately 6 million people are affected by cornea-related vision impairment and blindness globally, with infectious keratitis (IK) being the major cause in both developed and developing countries (Ung et al., 2019; Stapleton, 2021; Ting et al., 2021e). IK is a painful and sight-threatening condition that often requires intensive medical and/or surgical interventions. Several risk factors have been implicated in IK, including contact lens wear, trauma, ocular surface disease, and post-corneal surgeries, amongst others (Edwards et al., 2009; Khoo et al., 2019; Ting et al., 2021a; Ting et al., 2021b).

Post-keratoplasty infectious keratitis (PKIK) represents a unique clinical entity that frequently poses diagnostic and therapeutic challenges (Song et al., 2021). The incidence of PKIK varies considerably depending on the geographical areas where a higher incidence of PKIK was reported in developing countries (up to 11.9%) than developed countries (up to 7.9%) (Edelstein et al., 2016; Chen et al., 2017; Okonkwo et al., 2018; Sharma et al., 2021; Song et al., 2021) The manifestation of PKIK can be predisposed by a range of risk factors, including suture-related issues, ocular surface disease, failed grafts, recurrence of original infection, neurotrophic keratopathy, and persistent epithelial defects (Lin et al., 2016; Chen et al., 2017; Okonkwo et al., 2018; Griffin et al., 2020; Song et al., 2021; Dave et al., 2022), highlighting the importance of postoperative care, management of pre-existing ocular co-morbidities, and patient counselling/education.

Penetrating keratoplasty (PKP) has historically been the most commonly performed type of keratoplasty, though there has been a paradigm shift to lamellar keratoplasty such as endothelial keratoplasty (EK) and deep anterior lamellar keratoplasty (DALK), largely attributed to the lower risk of immunological graft rejection, improved visual outcome and faster recovery (Ting et al., 2012; Akanda et al., 2015; Park et al., 2015; Ting et al., 2023). This shifting pattern in keratoplasty not only influences the causes and characteristics of PKIK but also results in new types of post-keratoplasty complications such as interface infectious keratitis (IIK), a type of challenging IK that occurs along the surgically created graft-host interface plane in lamellar keratoplasty (Ting et al., 2019b; Sharma et al., 2021; Song et al., 2021). Gram-positive bacteria, particularly *Staphylococcus aureus* and *Streptococcus pneumoniae*, are the most often identified bacteria responsible for PKIK after PKP (Moorthy et al., 2011; Sun et al., 2017; Okonkwo et al., 2018; Griffin et al., 2020; Song et al., 2021; Dave et al., 2022), whereas fungi such as *Candida* species are particularly associated with PKIK after EK, predominantly due to donor graft-transmitted infection (Edelstein et al., 2016; Lin et al., 2016; Griffin et al., 2020; Song et al., 2021).

PKIK can often result in significant ocular morbidities, including poor visual outcome, graft rejection, graft failure, and, less frequently, endophthalmitis, if it is not managed promptly (Lin et al., 2016; Ting et al., 2019a; Sharma et al., 2021; Song et al., 2021). Studies have shown that 70-80% patients ended up with a Snellen visual acuity of <6/60 after PKIK (Wagoner et al., 2007; Chen et al., 2017). Graft failure may occur up to 71.4% of PKIK cases, in which older grafts are more susceptible (Chen et al., 2017; Song et al., 2021). These issues highlight the negative impact of PKIK on the affected patients and can exacerbate the supply issue with donor corneas.

Over the past decade, only two studies in the UK have specifically examined the causes and/or management and outcomes of PKIK (Okonkwo et al., 2018; Griffin et al., 2020). In view of the existing gap in the literature and the significant impact of PKIK on affected patients and healthcare systems, this study aimed to examine the clinical characteristics, risk factors, outcomes, and prognostic factors of PKIK within a major tertiary hospital in the UK.

## Materials and methods

This was a retrospective observational study of all patients who presented with IK to the Queen’s Medical Centre, Nottingham, UK, between September 2015 and August 2022 (a 7-year study period). This study was approved by the Clinical Governance team of the Nottingham University Hospitals NHS Trust as a clinical audit (Ref: 19-265C) and was conducted in accordance with the tenets of Declaration of Helsinki.

### Case identification

**Figure 1:**
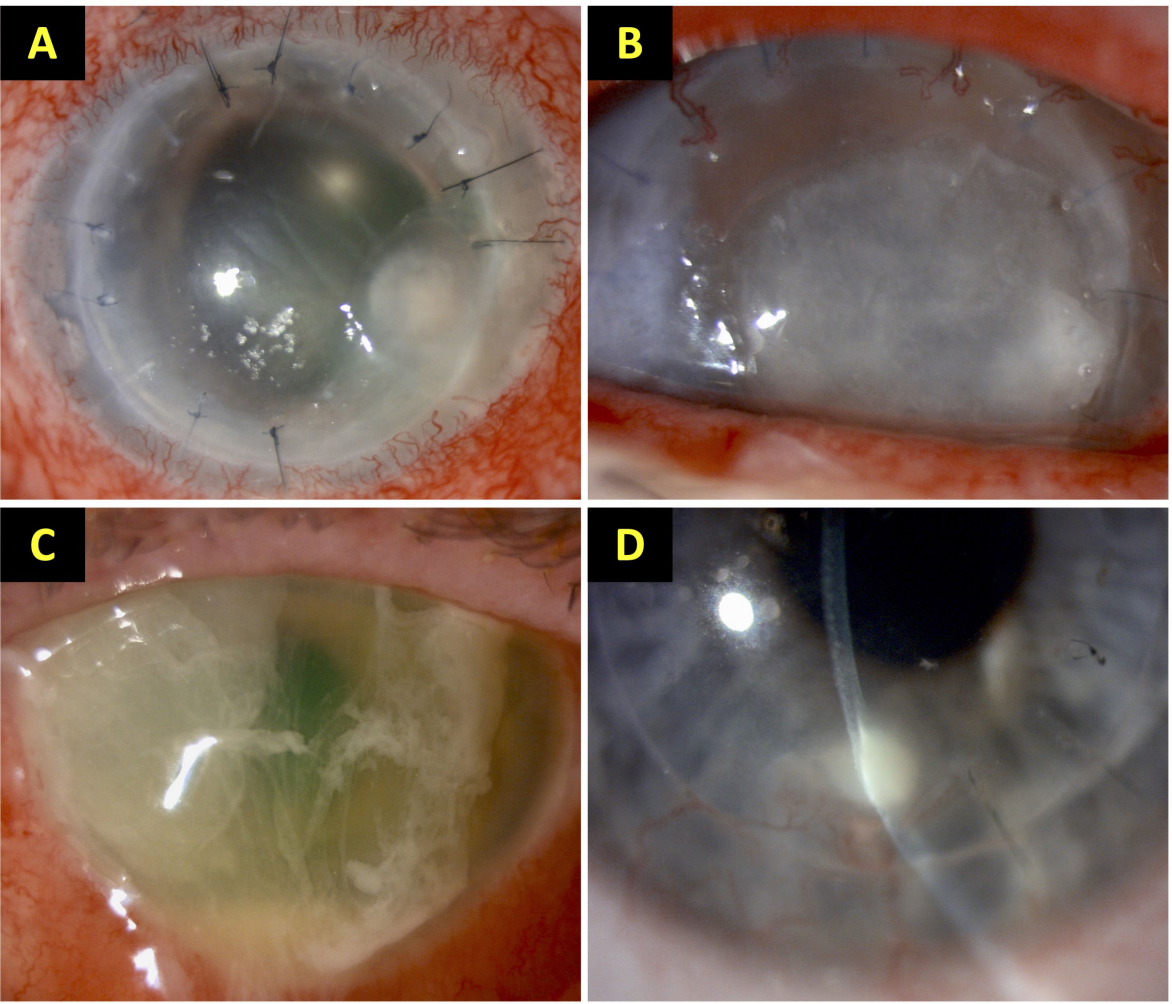
Examples of post-keratoplasty infectious keratitis (PKIK). **(A)** A case of suture-related PKIK caused by *Staphylococcus aureus* in an eye after penetrating keratoplasty. **(B)** A case of PKIK caused by *Moraxella catarrhalis* in an eye with failed penetrating keratoplasty with bullous keratopathy, while on topical steroids. **(C)** A case of PKIK caused by *Pseudomonas aeruginosa* in an eye with failed Descemet stripping automated endothelial keratoplasty with bullous keratopathy, while on topical steroids. **(D)** A case of graft-host interface infectious keratitis after deep anterior lamellar keratoplasty (using manual dissection technique) for keratoconus. These figures were adapted from Song et al. study (Song et al., 2021).

All potential IK cases, including those caused by bacteria, fungi and parasites, were initially identified from the local microbiological database, which captured all the cases that had undergone corneal sampling for presumed IK at the QMC, Nottingham, UK (Ting et al., 2021d). Both culture-positive and culture-negative (i.e. no growth on any culture media) IK cases were included. Culture-negative IK were diagnosed based on positive clinical findings (e.g. presence of corneal ulceration, infiltrate and/or anterior chamber inflammatory activity) and the clinical course of the disease where improvement and/or resolution of the infection was achieved by intensive topical antimicrobial therapy. Subsequently, medical case notes were reviewed to identify eligible patients for the study (i.e. those who had previously undergone keratoplasty and developed PKIK). All types of keratoplasty, including PKP, DALK and EK, were included and analyzed (**Figures 1A–D**).

### Data collection

Relevant data, including demographic factors, risk factors, clinical characteristics, causative microorganisms, details of keratoplasty [including the original indication, age of the most recent graft at presentation, presence of corneal sutures, use of topical steroids, health of the graft, and location of infection (e.g. surface or graft-host interface)], corrected-distance-visual-acuity (CDVA), management, outcome, and complications, were collected using a standardized excel proforma. Cases without complete initial/follow-up data or not related to infection were excluded from this study. Similar to the previous study (Ting et al., 2021a), the size of epithelial defect and infiltrate were categorized as small (<3mm), moderate (3.1-6mm), or large (>6mm), whereas the ulcer location was divided into central (any involvement of the visual axis), paracentral (in between the central and peripheral location), or peripheral location (the entire ulcer was within 3mm from the limbus)

### Clinical management

The clinical management of IK had been detailed in the previous studies (Ting et al., 2021a; Ting et al., 2021c). As per local guidelines, all patients underwent corneal sampling for microbiological investigations if one of the following criteria was met: (1) diameter of ulcer ≥1mm; (2) central location; (3) significant anterior chamber reaction or hypopyon; and/or (4) atypical clinical presentation. Corneal samples were sent for microscopic examination (with Gram staining), culture and/or polymerase chain reaction (PCR), and sensitivity testing. Corneal samples were inoculated on a chocolate agar (for fastidious bacteria), a blood agar, a fastidious anerobic agar, and a Sabouraud dextrose agar (for fungi). For suspected cases of Acanthamoeba keratitis, corneal swab and/or epithelial biopsy was obtained for culture on non-nutrient agar with *Escherichia coli* overlay or for PCR (Wong et al., 2023). All culture agar plates were incubated for at least 1 week (and up to 3 weeks for suspected Acanthamoeba keratitis and fungal keratitis). In vivo confocal microscopy (IVCM), using the Heidelberg Retinal Tomography (HRT) II with Rostock Cornea Module (Heidelberg Engineering Ltd, Hertfordshire, UK), was also performed in some culture-negative cases if fungal or Acanthamoeba keratitis was suspected.

Patients diagnosed with PKIK were treated with intensive topical fluoroquinolone monotherapy or topical fortified dual therapy, consisting of a cephalosporin and an aminoglycoside or fluoroquinolone. The initial choice of treatment was guided by the severity and the clinicians’ preference. Hospitalization was indicated if the ulcer was severe or unresponsive to initial antibiotic treatment, or if the patient was unable or unlikely to comply with the intensive treatment regimen. Based on the clinical course, causative organism(s), antimicrobial susceptibility results, and treatment response, antimicrobial therapy was adjusted as needed.

### Statistical analysis

Statistical analysis was performed using SPSS version 28.0 (IBM SPSS Statistics for Windows, Armonk, NY, USA). All continuous data were presented as mean ± standard deviation (SD) and/or 95% confidence interval (CI). Comparisons between groups were conducted using Pearson’s Chi square or Fisher’s Exact test (when >20% cells have expected frequencies of <5) where appropriate for categorical variables, and T test or Mann-Whitney U test for continuous variables.

The main outcome measures were CDVA, complete corneal healing time (defined as the time taken from initial presentation to complete resolution of infection with corneal epithelialization), and graft status. For analytic purposes, Snellen vision was converted to logMAR vision. Vision of counting fingers (CF), hand movement (HM), perception of light (PL) and no perception of light (NPL) were quantified as 1.9 logMAR, 2.3 logMAR, 2.8 logMAR and 3.0 logMAR respectively (Lange et al., 2009). Logistic regression analysis was performed in patients who had functioning corneal graft before the presentation of IK to examine for any potential predicting factors for poor visual outcome [i.e., CDVA of <6/60 (or <1.0 logMAR) and poor corneal healing (i.e., >30 days taken for complete healing).

Kaplan-Meier survival analysis was performed to estimate graft survival/failure until the last follow-up, and log-rank test was used to examine the difference (if any) among PKP, DALK and EK. Graft failure was defined by the presence of irreversible corneal endothelial failure or significant corneal opacity affecting the vision. Univariable and multivariable Cox proportionate hazard regression analysis was performed to determine potential prognostic factors for graft failure following PKIK within 5 years of follow-up after PKIK and the results were reported in hazard ratio (HR).

## Results

### Baseline characteristics

A total of 49 cases of PKIK were identified during the study period. The mean age was 64.6 ± 20.4 years and 49% were female patients (**Table 1**). Patients presented with PKIK following EK were significantly older than those with PK and DALK (p<0.001). At the initial presentation of PKIK, the mean CDVA was 1.69 ± 0.99 logMAR and 34 (69.4%) cases had a CDVA of <1.0 logMAR. The majority of PKIK cases were presented with small epithelial defect (25, 51.0%), small infiltrate (24, 49.0%), and paracentrally located infection (24, 49.0%). Hypopyon was detected in 14 cases (28.6%) cases. When compared to PKP and EK cases, patients presented with DALK-related PKIK had a significantly better presenting CDVA (p=0.001), smaller epithelial defect (p=0.038), smaller infiltrate (p=0.003), and absence of hypopyon (p=0.038).

**Table 1:** An overview of the baseline characteristics of post-keratoplasty infectious keratitis based on the types of keratoplasty, including penetrating keratoplasty (PK), deep anterior lamellar keratoplasty (DALK) and endothelial keratoplasty (EK).

Parameters	Total N = 49 (%)	PK N = 24 (%)	DALK N = 11 (%)	EK N = 14 (%)	P-value*
Age, years	64.6 ± 20.4	63.1 ± 20.4	47.2 ± 12.0	81.0 ± 12.1	<0.001
Female gender	24 (49.0)	12 (50.0)	4 (36.4)	8 (57.1)	0.58
Laterality (left eye)	27 (55.1)	14 (58.3)	4 (36.4)	9 (64.3)	0.34
Presenting CDVA, logMAR					0.001
≥0.6	11 (22.4)	3 (12.5)	7 (63.6)	1 (7.1)	
<0.6 – ≥1.0	5 (10.2)	1 (4.2)	0 (0.0)	4 (28.6)	
<1.0	33 (67.3)	20 (83.3)	4 (36.4)	9 (64.3)	
Size of epithelial defect					0.038
No or small (<3mm)	28 (57.1)	14 (58.3)	9 (81.8)	4 (28.6)	
Moderate (3-6mm)	12 (24.5)	4 (16.7)	2 (18.2)	7 (50.0)	
Large (>6mm)	9 (18.4)	6 (25.0)	0 (0.0)	3 (21.4)	
Size of infiltrate					0.003
No or small (<3mm)	27 (55.1)	14 (58.3)	10 (90.9)	3 (21.4)	
Moderate (3-6mm)	10 (20.4)	3 (12.5)	0 (0.0)	7 (50.0)	
Large (>6mm)	12 (24.5)	7 (29.2)	1 (9.1)	4 (28.6)	
Location					0.39
Central	19 (38.8)	10 (41.7)	3 (27.3)	6 (42.9)	
Paracentral	24 (49.0)	10 (41.7)	7 (63.6)	7 (50.0)	
Peripheral	6 (12.2)	4 (16.7)	1 (9.1)	1 (7.1)	
Presence of hypopyon	14 (28.6)	7 (29.2)	0 (0.0)	7 (50.0)	0.038
Latest graft indication					<0.001
* Failed graft*	13 (26.5)	9 (37.5)	1 (9.1)	4 (28.6)	
* Keratoconus*	8 (16.3)	2 (8.3)	6 (54.5)	0 (0.0)	
* FECD*	8 (16.3)	0 (0.0)	0 (0.0)	8 (57.1)	
* Infectious keratitis*	4 (8.2)	3 (12.5)	1 (9.1)	0 (0.0)	
* Bullous keratopathy*	10 (20.4)	7 (29.2)	0 (0.0)	2 (14.3)	
*Others*	6 (12.2)	3 (12.5)	3 (27.3)	0 (0.0)	
Age of graft, years					0.013
<1	15 (30.6)	6 (25.0)	1 (9.1)	8 (57.1)	
1-2	8 (16.3)	4 (16.7)	4 (36.4)	0 (0.0)	
2-5	11 (22.4)	3 (12.5)	4 (36.4)	4 (28.6)	
>5	15 (30.6)	11 (45.8)	2 (18.2)	2 (14.3)	
Existing graft failure	18 (36.7)	11 (45.8)	0 (0.0)	7 (50.0)	0.016
Predisposing factors^#^					0.026
Suture-related issues	15 (30.6)	10 (41.7)	5 (45.5)	0 (0.0)	
Bullous keratopathy	18 (36.7)	11(45.8)	0 (0.0)	7 (50.0)	
Ocular surface disease	18 (36.7)	10 (41.7)	3 (27.3)	5 (35.7)	
Contact lens wear	5 (10.2)	2 (8.3)	2 (18.2)	1 (7.1)	
Immunosuppression^$^	4 (8.2)	4 (16.7)	0 (0.0)	0 (0.0)	
Trauma	3 (6.1)	3 (12.5)	0 (0.0)	0 (0.0)	
Existing use of topical steroids	25 (51.0)	9 (37.5)	6 (54.5)	10 (71.4)	0.13

FECD, Fuchs endothelial corneal dystrophy; CDVA, Corrected-distance-visual-acuity.

Continuous values are presented as mean ± standard deviation.

*Statistical comparisons are made between PK, DALK and EK, using Chi-square, Fisher exact and ANOVA tests where appropriate. Significant values are underlined.

^#^Some cases have more than 1 predisposing factor.

^$^Immunosuppression includes the use of systemic immunosuppressive drugs and diabetes mellitus.

### Causative organisms and antimicrobial susceptibility results

Of all 49 cases of PKIK, 30 (61.2%) cases were culture positive (n=31 organisms), including one case of poly-bacterial infection. Bacteria were isolated in most cases (29, 96.7%) and fungus was isolated in 1 (3.3%) case (**Table 2**). *Staphylococcus aureus* (10, 33.3%) was the most commonly isolated bacteria, followed by *Streptococcus pneumoniae* (4, 13.3%) and *Pseudomonas aeruginosa* (4, 13.3%). The only case of poly-bacterial infection, which occurred after an EK, was caused by coagulase-negative staphylococcus and *Propionibacterium* spp. *S. aureus* was the most common causative organism in PKP and DALK whereas coagulase-negative Staphylococci (CoNS) was the most common causative organism in EK. *Candida parapsilosis* was the only identified fungal infection, which occurred in a case of PKP. Out of the 19 culture-negative cases, 16 (84.2%) cases were treated as presumed bacterial infection, 2 (10.5%) were treated as presumed fungal infection (based on IVCM findings), and 1 (5.3%) was treated as mixed bacterial and fungal infection. There was no case of Acanthamoeba PKIK noted in this study.

**Table 2:** Causative organisms of post-keratoplasty infectious keratitis based on the types of keratoplasty, including penetrating keratoplasty (PK), deep anterior lamellar keratoplasty (DALK), and endothelial keratoplasty (EK).

Microbiological results	Total N (%)	PK N (%)	DALK N (%)	EK N (%)	P-value
Culture results (total)	49 (100)	24 (100)	11 (100)	14 (100)	0.21
Positive	30 (61.2)	12 (50.0)	7 (63.6)	11 (78.6)	
Negative	19 (38.8)	12 (50.0)	4 (36.4)	3 (21.4)	
Organisms (total)	31 (100)	12 (100)	7 (100)	12 (100)	0.13**
Gram-positive bacteria	22 (71.0)	6 (50.0)	7 (100)	9 (75.0)	
* Staphylococcus aureus*	10 (32.3)	5 (41.7)	5 (71.4)	0 (0)	
* CoNS*	5 (16.1)	0 (0)	1 (14.3)	4 (33.3)*	
* Streptococcus pneumoniae*	4 (12.9)	0 (0)	1 (14.3)	3 (25.0)	
* Propionibacterium spp.*	2 (6.5)	1 (8.3)	0 (0)	1 (8.3)*	
* Corynebacterium spp.*	1 (3.2)	0 (0)	0 (0)	1 (8.3)	
Gram-negative bacteria	8 (25.8)	5 (41.7)	0 (0)	3 (25.0)	
* Pseudomonas aeruginosa*	4 (12.9)	2 (16.7)	0 (0)	2 (16.7)	
* Moraxella catarrhalis*	3 (9.7)	3 (25.0)	0 (0)	0 (0)	
* Serratia marcescnes*	1 (3.2)	0 (0)	0 (0)	1 (8.3)	
Fungi					
* Candida parapsilosis*	1 (3.2)	1 (8.3)	0 (0)	0 (0)	

CoNS, Coagulase negative Staphylococci.

*One case isolated two organisms.

**Comparison of types of organism (i.e., Gram-positive bacteria, Gram-negative bacteria and fungi) among PK, DALK and EK.

Gram-positive bacteria exhibited good-to-excellent susceptibility to cephalosporin (100%), fluoroquinolone (66.7%-73.3%), and aminoglycoside (73.3%-100%), whereas Gram-negative bacteria showed excellent susceptibility to cephalosporin (100%), fluoroquinolone (80.0%-100.0%), and aminoglycoside (100.0%). Details of the antimicrobial susceptibility results of all 30 causative bacteria are summarized in **Table 3**.

**Table 3:** Summary of antibiotic susceptibility of causative bacteria of post-keratoplasty infectious keratitis in Nottingham, UK, between 2015 and 2022.

Bacteria	Antibiotics					
	Penicillin^#^	Cefuroxime	Amikacin	Gentamicin	Ciprofloxacin	Levofloxacin
**Gram-positive**	16/22 (80.0)	2/2 (100.0)	1/1 (100.0)	11/15 (73.3)	11/15 (73.3)	4/6 (66.7)
* Staphylococcus spp.*	9/15 (60.0)	2/2 (100.0)	1/1 (100.0)	11/15 (73.3)	10/15 (66.7)	0/2 (0.0)
* Streptococcus pneumoniae*	4/4 (100.0)	0	0	0	0	4/4 (100.0)
Others *	3/3 (100.0)	0	0	0	1/1 (100.0)	0
**Gram-negative**	0/2 (0.0)	1/1 (100.0)	5/5 (100.0)	5/5 (100.0)	4/5 (80.0)	2/2 (100.0)
* Pseudomonas aeruginosa*	0	0	4/4 (100.0)	4/4 (100.0)	3/4 (75.0)	0
Others **	0/2 (0.0)	1/1 (100.0)	1/1 (100.0)	1/1 (100.0)	1/1 (100.0)	2/2 (100.0)

^#^Penicillin group includes penicillin, amoxicillin, and flucloxacillin.

*Includes Propionibacterium spp. and Corynebacterium spp.

** Includes Moraxella catarrhalis and Serratia marcescens.

### Details of keratoplasty, types of infection and predisposing factors

The majority of PKIK occurred after PKP (24, 49.0%), followed by EK (14, 28.6%) and DALK (11, 22.4%). The most common graft indications for PKP, DALK and EK were regraft (9, 37.5%), keratoconus (6, 54.5%) and Fuchs endothelial corneal dystrophy (FECD; 8, 57.1%), respectively (**Table 1**). The mean interval of the latest graft and the initial presentation of PKIK was 37.3 ± 46.2 months, with 15 (31.3%) grafts presenting within a year of keratoplasty. There was a significant difference in the proportion of PKIK presenting within a year among EK (8, 57.1%), PKP (6, 25.0%), and DALK (1, 9.1%) (p=0.013). Eighteen (36.7%) grafts had failed before the presentation of PKIK, mainly occurring in PKP (11, 45.8%) and EK (7, 50.0%) cases.

The majority (45, 91.8%) of cases were related to ocular surface infection whereas 4 (8.2%) cases were related to interface infectious keratitis (IIK), including 3 DALK cases and 1 EK case. Bullous keratopathy (18, 36.7%), ocular surface diseases (OSD; 18, 36.7%), suture-related complications (15, 30.6%), and contact lens wear (5, 10.2%) were the most common predisposing factors for PKIK. Concurrent use of topical corticosteroids was noted in 25 (51.0%) PKIK cases. Suture-related PKIK occurred in 10 (66.7%) and 5 (33.3%) cases of PKP and DALK, respectively. Of these 15 suture-related cases, 6 (40%), 4 (26.7%), 3 (20.0%), and 2 (13.3%) of the PKIK occurred at <1 year, 1-2 years, 2-5 years, and >5 years post-keratoplasty, respectively.

### Management and clinical outcomes

Hospitalization for intensive antimicrobial therapy was necessary in 33 (67.3%) cases. The majority (36, 73.5%) of the cases were successfully managed with topical antimicrobial therapy alone (**Table 4**). Surgical/procedural interventions were required for 13 (26.5%) cases, including regrafting (6, 12.2%), corneal gluing (5, 10.2%), amniotic membrane transplantation (AMT; 2, 4.1%), tarsorrhaphy (2, 4.1%), intrastromal antibiotic injection (1, 2.0%), and intravitreal antibiotic injection for suspected but non-proven endophthalmitis (1, 2.0%). Among the 6 cases that required a regraft, 4 cases (all EK) had a failed graft prior to the PKIK presentation whereas 2 cases (1 PKP and 1 DALK) had a functioning graft prior to PKIK. All 6 cases underwent elective optical PKP, including the 4 EK cases that were originally awaiting a repeat EK.

**Table 4:** Clinical management of post-keratoplasty infectious keratitis, categorized by functioning and failed grafts at the initial presentation.

Management	Total (N = 49)	Functioning Grafts (N = 31)	Failed Grafts (N = 18)	P-value
Hospital Admission	33 (67.3)	22 (70.9)	11 (61.1)	0.48
Topical antimicrobial therapy alone	36 (73.5)	25 (80.6)	11 (61.1)	0.14
Surgical/procedural interventions	13 (26.5)	6 (19.4)	7 (38.8)	0.14
Corneal gluing	5 (10.2)	4 (12.9)	1 (5.5)	
Amniotic membrane transplantation	2 (4.1)	1 (3.2)	1 (5.6)	
Tarsorrhaphy	2 (4.1)	1 (3.2)	1 (5.6)	
Keratoplasty (optical)	6 (12.2)	2 (6.5)	4 (22.2)	
Intrastromal / intravitreal injections	2 (4.1)	1 (3.2)	1 (5.5)	

**Figure 2:**
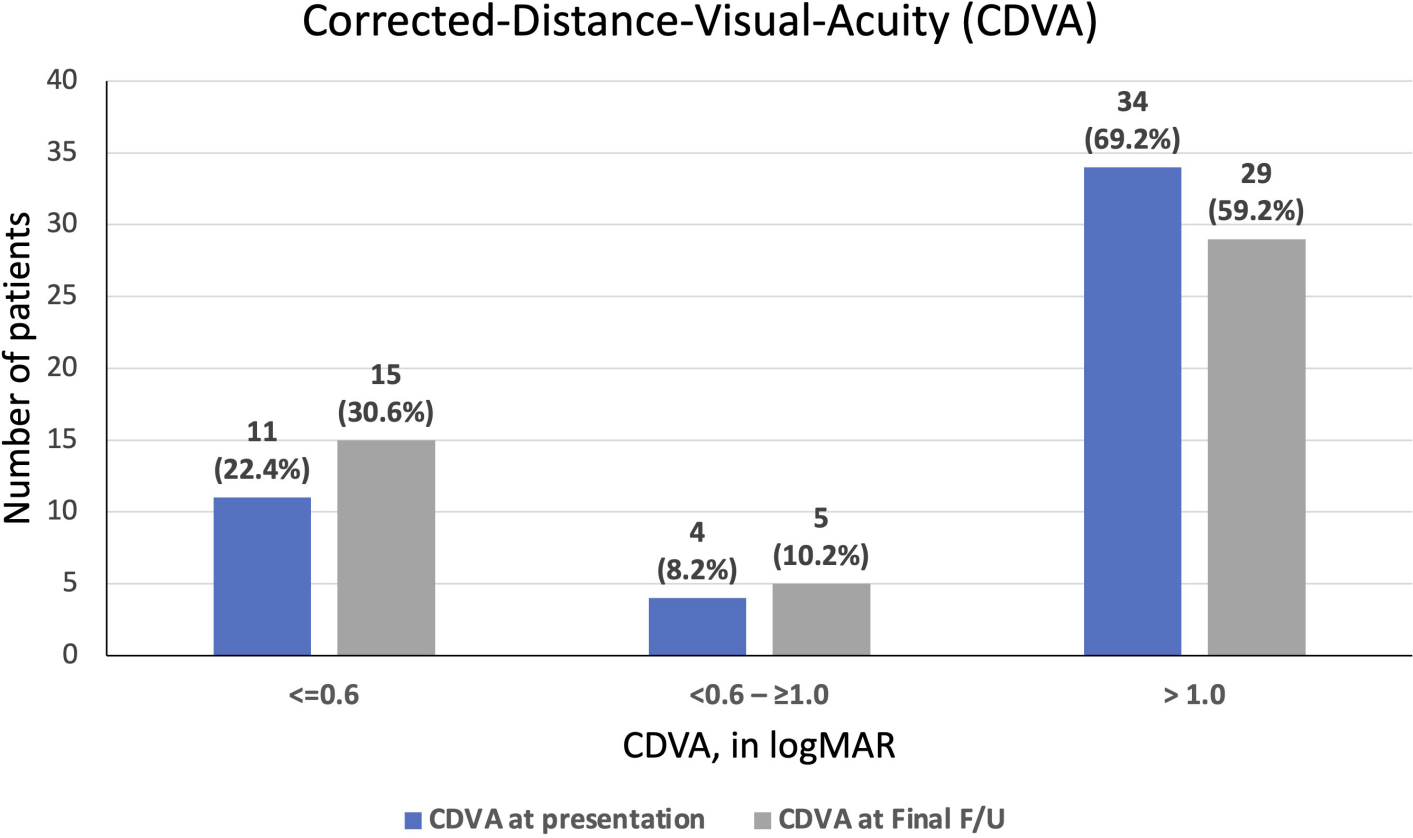
A summary of corrected-distance-visual-acuity (CDVA) at presentation of post-keratoplasty infectious keratitis (PKIK) and at final follow-up. Note the increased proportion of patients achieving a CDVA of ≥ 0.6 logMAR and the decreased proportion of patients achieving CDVA of <1.0 logMAR from baseline to final follow-up after PKIK.

At a mean follow-up of 20.1 ± 20.1 months, the overall final CDVA was 1.50 ± 0.99 logMAR, with 24 (50%) patients achieving visual improvement and 15 patients (31.3%) having a worse final CDVA (**Figure 2**). The mean corneal healing time was 2.3 ± 2.4 months. Of the 31 cases with functioning grafts at presentation, 12 (38.7%) grafts failed by the last follow-up visit (a mean follow-up duration of 24.1 ± 22.6 months) and only 15 (48.4%) patients retained a CDVA of ≥1.0 logMAR. Among the 12 cases of *de novo* graft failure after PKIK, significant corneal opacity and irreversible endothelial failure were noted as the primary reason for failure in 8 (66.7%) and 4 (33.3%) cases, respectively. Significant corneal opacity was noted in 4 PKP, 3 DALK and 1 EK cases whereas irreversible endothelial failure occurred in 2 PKP and 2 EK cases.

**Figure 3:**
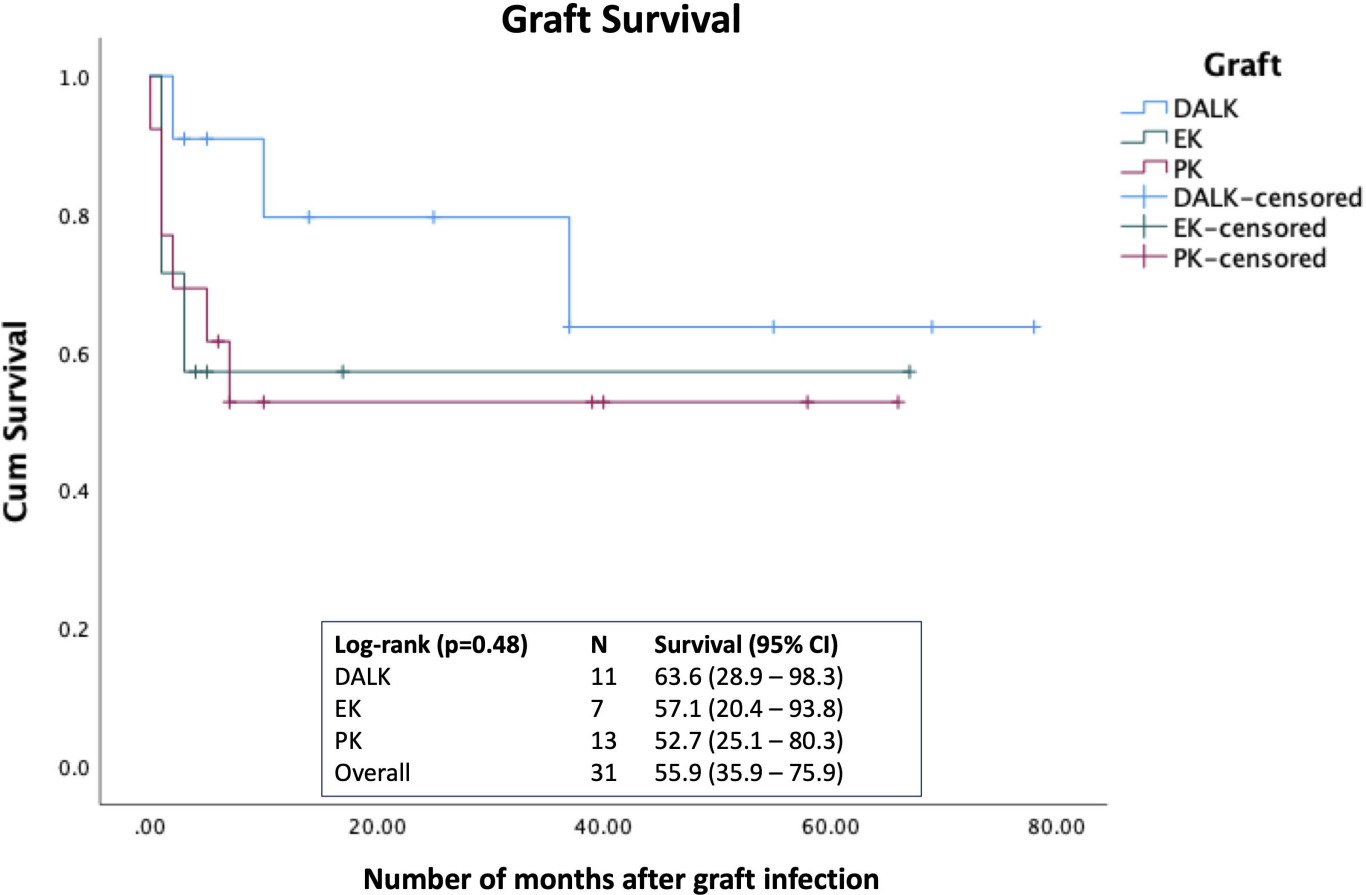
Kaplan-Meier survival curve demonstrating the graft survival rate following post-keratoplasty infectious keratitis (PKIK). Log-rank test was used to examine the difference in the 5-year graft survival rate among penetrating keratoplasty (PK), deep anterior lamellar keratoplasty (DALK), and endothelial keratoplasty (EK).

The overall estimated 5-year survival rate post-PKIK was 55.9% (95% CI, 35.9%-75.9%), with DALK having the highest survival rate [63.6% (95% CI, 28.9%-98.3%)], followed by EK [57.1% (95% CI, 20.4%-93.8%)] and PKP [52.7% (95% CI, 25.1%-80.3%)], though no statistical difference was observed (p=0.48; **Figure 3**). Univariable Cox regression analysis demonstrated presenting CDVA, infiltrate size, ulcer location, and hypopyon as significant hazards of graft failure, though infiltrate size >3mm [HR 8.10 (95% CI, 1.31-49.93); p=0.024] was the only significant hazard of graft failure within 5 years of PKIK in the multivariable Cox regression model (**Table 5**).

**Table 5:** Multivariable Cox proportional hazard regression analysis for graft failure within 5 years of post-keratoplasty infectious keratitis (PKIK).

Parameters	Univariable		Multivariable*	
	HR (95% CI)	P-value	HR (95% CI)	P-value
Age > 60 years	2.72 (0.81 – 9.18)	0.11	–	–
Male gender	1.14 (0.36 – 3.56)	0.83	–	–
Presenting CDVA <6/60	8.74 (1.12 – 68.24)	0.039	4.95 (0.47 – 52.34)	0.18
Infiltrate size >3mm	11.99 (3.04 – 47.26)	<0.001	8.10 (1.31 – 49.93)	0.024
Central ulcer	6.24 (1.83 – 21.29)	0.003	2.27 (0.51 – 10.13)	0.28
Presence of hypopyon	4.29 (1.19 – 15.42)	0.026	2.13 (0.52 – 8.66)	0.29
Positive culture results	0.82 (0.27 – 2.55)	0.73	–	–
Age of graft, years		0.82	–	–
<1	Reference group	–		
1-2	0.53 (0.10 – 2.81)	0.46		
2-5	0.99 (0.24 – 4.23)	0.99		
>5	0.57 (0.11 – 2.96)	0.50		
Use of topical steroids	0.99 (0.32 – 3.12)	0.99	–	–

HR, Hazard ratio.

*Only factors with p<0.1 during the univariate analysis are included in the Multivariable logistic regression analysis was performed. Significant p-values are underlined. -, not mentioned.

## Discussion

PKIK represents a challenging clinical entity which can significantly affect the vision and result in graft failure despite appropriate management. We observed a significant difference in the presenting clinical features, risk factors and onset of infection (i.e., the interval between keratoplasty and PKIK) among PKP-, DALK- and EK-related PKIK. We also observed poor visual and graft survival outcomes following PKIK, with ~40% previously functioning grafts culminating in graft failure (within 5 years) and ~50% having a CDVA of <1.0 logMAR, highlighting a significant impact of PKIK on the affected patients.

### Clinical characteristics and risk factors

PKIK can affect individuals of various ages, ranging from 1 to 95 years (Vajpayee et al., 2002; Sung et al., 2015; Lin et al., 2016; Okonkwo et al., 2018; Dohse et al., 2020; Griffin et al., 2020; Song et al., 2021; Sati et al., 2022). In our study, we observed that PKIK patients who underwent EK were significantly older (mean of 81 years old) compared to those who had PKP (mean of 63 years old) or DALK (mean of 47 years old), which was consistent with Dohse et al. study (Dohse et al., 2020). The more elderly age of EK patients can be attributed to the predominant indication for EK in our study, namely FECD (57.1%), which tends to occur more frequently in older populations (Soh et al., 2020). In contrast, DALK was mainly performed for keratoconus, which typically occurs in younger populations (Gadhvi et al., 2019). Hence the correlation with age in each keratoplasty group is likely to be associated with the underlying graft indications.

Suture-related complications have consistently been identified as the leading cause of PKIK, with reported rates ranging from 20% to 50% in previous studies (Huang et al., 2000; Lin et al., 2016; Chen et al., 2017; Dohse et al., 2020; Griffin et al., 2020; Song et al., 2021). Our study aligns with these findings as we observed suture-related issues in 30.6% of our PKIK cases. The presence of broken or loose sutures can result in a breach in the corneal epithelium, which represents one of the major ocular surface defense mechanisms. In addition, the exposure of loose or broken sutures traps mucus and provides a source for biofilm formation, which increases microbial resistance to antimicrobial therapy (Song et al., 2021). Therefore, many authors have recommended early suture removal in appropriate cases to reduce the risk of PKIK (Christo et al., 2001; Gnanaraj et al., 2007; Okonkwo et al., 2018; Song et al., 2021). This has to be balanced with the risk of inducing high astigmatism from removal of all sutures in eyes where the astigmatic error, with a variable number of remaining sutures, is managed with glasses and/or contact lenses with good vision.

Interestingly, bullous keratopathy (36.7%) and OSD (36.7%) were shown to be the most common predisposing factors in our study. This is partly attributed to the paradigm shift from PKP to EK for corneal endothelial diseases where sutures are not/minimally required. Moreover, ‘clean’ cases are selected out for DALK or EK, leaving the relatively higher risk cases for PKP. OSD has also been recognised as a significant risk factor of PKIK in previous studies, with rates ranging from 20% to 60% (Huang et al., 2000; Lin et al., 2016; Chen et al., 2017; Song et al., 2021). OSD can compromise the quantity and quality of the tear film, innate host defense mechanisms, epithelial integrity and homeostasis, and wound healing (in those with neurotrophic keratopathy), all of which can heighten the risk of PKIK (Akpek and Gottsch, 2003; Dua et al., 2018; Ting et al., 2022b). We also observed that 4 cases of PKIK that were initially awaiting a repeat EK before the infection had to be converted to a PKP following the infection. The continual rise in such clinical scenario is anticipated in the future in view of the increasing waiting time of keratoplasty secondary to a global shortage of donor corneas (Gain et al., 2016). This suggests the potential benefit of prophylactic use of topical antibiotics in patients with bullous keratopathy or OSD while awaiting a repeat EK.

### Interface infectious keratitis

IIK is a unique type of PKIK in which the infection occurs at the graft-host interface. The deep-seated nidus of infection presents significant diagnostic and therapeutic challenges as there is a lack of direct access for corneal sampling of the infection and considerable difficulty for topical and systemic antimicrobial drugs to effectively reach the site of infection (Fontana et al., 2019; Ting et al., 2019b; Song et al., 2021). It is commonly caused by *Candida* species, primarily due to graft-transmitted infection (Fontana et al., 2019). In a case series of 1088 Descemet stripping automated endothelial keratoplasty (DSAEK), the incidence of IIK was found to be only 0.92%, suggesting the rarity of this clinical entity (Nahum et al., 2014). A small cluster of post-EK fungal IKK/endophthalmitis has also been reported in Europe, with *Candida* spp. being the most common organisms (Lau et al., 2019). The use of hypothermic medium instead of organ culture has been implicated in the increased incidence of post-EK IIK. In the two recent UK PKIK studies (Okonkwo et al., 2018; Griffin et al., 2020), no reports of IIK were documented.

In our study, we observed four cases of IIK, with three cases related to DALK and one case related to EK. Fortunately, all four cases of IIK in our study responded well to topical and intrastromal antimicrobial treatment/injections without the need for surgical intervention. In two cases where culture results were negative, the diagnosis was aided by *in vivo* confocal microscopy (IVCM), which revealed potential fungal filaments and allowed for early initiation of antifungal treatment (Ting et al., 2019b; Ting et al., 2022a). Despite the challenges posed by IIK, all four grafts remained clear and functioning. Plausible explanations for the good visual outcomes observed in our study include timely diagnosis and treatment, the nature of the IIK and the absence of risk of endothelial rejection triggered by the infection (in DALK cases). All 4 IIK cases were related to an initial ocular surface/suture-related infection, which spread deeper and along the graft-host interface (as opposed to the IIK caused by graft-transmitted infection in EK cases).

### Causative organisms

Consistent with multiple previous studies, our findings demonstrated that Gram-positive bacteria were the most common causative organisms in PKIK (Tseng and Ling, 1995; Akova et al., 1999; Wagoner et al., 2007; Okonkwo et al., 2018; Griffin et al., 2020). *Staphylococcus aureus, Streptococcus pneumoniae, and coagulase-negative Staphylococcus* are known ocular commensals, which are often associated with OSD, a significant risk factor for PKIK (Vajpayee et al., 2002; Okonkwo et al., 2018; Griffin et al., 2020). The predominance of Gram-positive bacteria in PKIK underscores the importance of addressing ocular surface health and managing OSD as part of preventive strategies. However, it is worth noting that a Taiwanese study conducted by Chen et al. (2017) reported a higher incidence of *Pseudomonas aeruginosa* (38.1%) as the most commonly isolated organism in their cohort, which differs from our study where it accounted for only 12.9% of cases. This variation could be attributed to factors such as the use of contact lenses, warmer climate, and restricted first-line use of topical fluoroquinolones in Taiwan.

### Clinical outcomes

Our study demonstrated a poor visual outcome following PKIK, evidenced by a high proportion (59%) of patients who had a final CDVA of ≤1.0 logMAR. This is consistent with many other studies where poor visual outcome was observed. According to Chen et al. (2017), only 66.7% of their patients had a CDVA of <1.0 logMAR after recovering from PKIK. The figure is even higher (78.4%) according to a report by Wagoner et al. (2007)

Graft failure is a common and significant complication following PKIK, with reported rates varying from 7.3% to 71.4% in previous studies (Chen et al., 2017; Okonkwo et al., 2018; Dohse et al., 2020). Wagoner et al. (2007) reported an estimated 4-year graft survival rate of 35.8% after PKIK following PKP, which is lower than our estimated 5-year survival rate of 52.7% after PKP. The difference may be related to the different patient cohort, including different presenting severity of infection, indication and age of the graft, and the management. We also observed that DALK has the highest survival rate, though the superiority was not statistically significant, likely due to a type 2 error related to a small sample size. The lower incidence of PKIK and the higher chance of graft survival following PKIK observed in lamellar keratoplasty (Okonkwo et al., 2018; Dohse et al., 2020) further substantiate the advantages of lamellar keratoplasty over PKP. In addition, the risk of endothelial graft rejection/failure is eliminated in DALK cases. Our results also demonstrated that the severity of infection (i.e., infiltrate size of >3mm) was a significant hazard for graft failure. This highlights the importance of patient education and counselling on the importance of postoperative care following corneal transplantation to enable timely diagnosis and treatment of post-keratoplasty complications.

### Strengths and limitations

To the best of our knowledge, this study represents one of the largest studies in the UK over the past decade that specifically examined the clinical presentations, causes, outcomes and prognostic factors of PKIK. One of the study limitations is that we only included cases that had undergone corneal sampling for presumed IK; therefore, cases that were of mild severity might not have been captured by this study. However, we have a low threshold of performing corneal sampling in any patient presented with PKIK in our routine practice, evidenced by the high proportion (around 50%) of PKIK cases with mild severity (i.e., small epithelial defect/infiltrate) that underwent sampling and were included. Secondly, in contrast to some studies in the literature which focused only on PKIK after PKP (Moorthy et al., 2011; Chen et al., 2017; Sun et al., 2017), our study captured PKIK related to all types of keratoplasty, including PKP, DALK and EK, enabling meaningful comparisons of the clinical characteristics, risk factors, causes, and graft outcomes among the three groups. Interestingly, the two UK studies (in the past decade) did not observe/report any PKIK following EK. Our study also highlighted emerging cases of IIK in the UK and the potential role of IVCM for aiding the diagnosis for this type of deep-seated graft infection. It was beyond our scope to investigate the incidence of PKIK in different types of keratoplasty as we did not have data for all the keratoplasty performed in Nottingham. In addition, some of the PKIK cases included in this study had their keratoplasty performed elsewhere, which complicated the estimation of incidence of PKIK within Nottingham. An analysis of the UK national corneal transplant data would be invaluable in the future to determine the incidence and outcomes of PKIK in the UK on a larger scale, though the national database is limited by the lack of important clinical data such as the causative organisms, risk factors, clinical characteristics, and management of PKIK.

In conclusion, PKIK is a significant complication that frequently culminates in poor visual outcome and/or irreversible graft failure. Loose/broken sutures, bullous keratopathy, and ocular surface disorders are the main predisposing factors for PKIK. Based on these findings, we advocate for early suture removal (where possible), use of prophylactic topical antibiotics (in bullous keratopathy cases, especially in eyes with visual potential that are awaiting repeat endothelial keratoplasty), and optimization of the ocular surface health to reduce the risk of PKIK.

## Data availability statement

The original contributions presented in the study are included in the article/supplementary material. Further inquiries can be directed to the corresponding author.

## Ethics statement

This study was approved by the Clinical Governance team of the Nottingham University Hospitals NHS Trust as a clinical audit (Ref: 19-265C).

## Author contributions

Study conceptualization, design and supervision: DT. Data collection: ZO, TW, LS, YH, ML. Data analysis: ZO, DT. Data interpretation: ZO, DS, HD, DT. Drafting of initial manuscript: ZO, DT. Critical revision of manuscript: TW, LS, YH, ML, DS, HD. All authors contributed to the article and approved the submitted version.
